# Scaling-up through piloting: dual-track provider payment reforms in China’s health system

**DOI:** 10.1093/heapol/czac080

**Published:** 2022-09-14

**Authors:** Alex Jingwei He

**Affiliations:** Department of Asian and Policy Studies, The Education University of Hong Kong, 10 Lo Ping Road, Tai Po, New Territories, Hong Kong SAR, China

**Keywords:** Scaling-up, pilot, provider payment, DRG, experiment, China

## Abstract

Gaining wide prominence in the global health arena, scaling-up increases the coverage of health innovations emerging from pilots and experimental projects to a larger scale. However, scaling-up in the health sector should not follow a linear ‘pilot-diffusion’ pathway in order to better facilitate local adaptation and policy refinement. This paper puts forth ‘scaling-up through piloting’ as a distinctive pathway for the strategic management of scaling-up in the health sector. It analyses the recent development of provider payment reforms in China, focusing particularly on the ongoing pilot programmes, namely diagnosis-related groups (DRGs) and diagnosis-intervention packet (DIP), that are being piloted in a dual-track fashion since 2020. Data were drawn from extensive documentary analysis and 20 in-depth interviews with key stakeholders, including decision-makers and implementers. This paper finds that scaling-up through piloting helps Chinese policymakers minimize the vast uncertainties associated with complex payment reforms and maximize the local adaptability of provider payment innovations. This pathway has forged a phased implementation process, allowing new payment models to be tested, evaluated, compared and adjusted in a full spectrum of local contexts before national rollout. The phased implementation creates a ‘slower is faster’ effect, helping reduce long-term negative consequences arising from improperly managed scaling-up in a complex system. Error detection and correction and recalibration of new policy tools can support national-level policy refinement in a more robust and dynamic fashion. Several key factors have been identified as crucial for strategic scaling-up: necessary central steering, a pragmatic piloting design, strong technical capacity and effective policy learning mechanisms

**Key messages**

- Scaling-up innovations in the health sector should not be undertaken in a linear manner but require strategic planning and flexible management.
- Scaling-up through piloting offers a dynamic pathway that is conducive to both local adaptation and national policy refinement.
- China’s ongoing provider payment reform has created a ‘slower is faster’ effect through a phased scaling-up process.
- Necessary national-level steering, a pragmatic piloting design, capacity building and policy learning are key factors for strategic scaling-up in a complex system.

## Introduction

The notion of scaling-up has gained increasing currency in the global health arena as an approach to resolving the multitude of health problems in the vast developing world (Hanson *et al.*, 2010). Defined as ‘deliberate efforts to increase the impact of successfully tested health innovations so as to benefit more people and to foster policy and programme development on a lasting basis’ (WHO and ExpandNet, 2010), scaling-up increases the coverage of health innovations emerging from pilots and experimental projects to a larger scale (Simmons *et al.*, 2007). An implicit assumption underlying the scaling-up literature is that a specific innovation is deemed efficacious and thus scalable to a wider scope. Although the literature does stress the critical importance of adaptation (Simmons *et al.*, 2007; Gilson and Schneider, 2010), this assumption is still problematic for three reasons.

First, health systems are not only complicated but also complex (Bali, 2020). They have been increasingly recognized as ‘complex adaptive systems’ that involve numerous interconnected components interacting in complex and non-linear ways, and therefore, the consequences of scaling-up are often highly unpredictable (Paina and Peters, 2012; Husain, 2017). Hence, ‘putting all eggs into one basket’ in scaling-up may run significant risks, especially in large countries with significant subnational disparities. Second, considering scaling-up as a purely technical process is often too simplistic and fails to recognize the political dimension of such exercises (Simmons *et al.*, 2007). Many innovations—clinical pathways, pay-for-performance schemes and provider payment methods, for example—typically involve the alteration of professional behaviours and/or deep material interests. Payment reforms, in particular, are highly contentious (Bertoli and Grembi, 2017; Abiiro *et al.*, 2021). As a result, scaling-up must take into account the complex interest structure of key stakeholders and their possible behavioural feedback. Doing so in reality inherently entails a careful experimental approach that is able to realign all incentives towards desired goals. Third, even when looking at the technical aspect of scaling-up alone, proper adaptation requires a great deal of evidence-based ‘learning by doing’ and micro-level managerial capabilities rather than mechanical repetition of innovations (Gilson and Schneider, 2010; WHO and ExpandNet, 2010; Husain, 2017).

Hence, it is the central contention of this paper that scaling-up in health policy should not follow a linear ‘pilot-diffusion’ pathway, but instead, strategic scaling-up often has an experimental dimension that facilitates managerial flexibility and recursive learning. The piloting of policy innovations prior to wider rollout is increasingly popular in many countries (Jowell, 2003; Nair and Howlett, 2016; Husain, 2017). It is a governance tool aimed at assessing a policy as it is being implemented and at facilitating policy innovation, implementation and validation (Ettelt *et al.*, 2022). This paper bridges the scaling-up literature and the sizable literature on policy piloting and puts forth ‘scaling-up through piloting’ as a distinctive pathway for the strategic management of scaling-up in the health sector. This pathway is illustrated by the case of provider payment reforms in the Chinese health system in recent years. This paper argues that scaling up through piloting serves as a pertinent strategy when policymakers seek to adopt health system innovations in the face of high complexities. It allows policy refinement to be combined with early implementation and accelerates policy learning and capacity building, both of which are crucial in middle- and low-income countries.

Reforming its provider payment system has been high on the agenda of China’s ambitious national healthcare reform. Moving away from fee-for-service (FFS) had been advocated for almost two decades, but major nationwide steps did not materialize until a few years ago (Gu and Page-Jarrett, 2018; Yip *et al.*, 2019). Empowered by growing policy capacity and advanced digital infrastructure, China has embarked on a major scaling-up of case-based payment innovations. A prominent feature of this reform is ‘scaling-up through piloting’, characterized by a dual-track piloting programme of two payment mechanisms: the globally renowned diagnosis-related groups (DRGs) and the domestically developed diagnosis-intervention packet (DIP). This scaling-up programme has been supported by strong central steering, a pragmatic piloting design, necessary technical capacity and a host of policy learning mechanisms.

This paper argues that scaling-up through piloting helps Chinese health policymakers minimize the vast uncertainties associated with complex payment reforms and maximize the local adaptability of provider payment innovations in the context of significant disparities across regions and health facilities. This study echoes the emerging wisdom in global health policy that scaling-up is not merely about technology transfer or innovation dissemination, but that a sound pathway for sustainable scaling-up innately requires strategic planning and adaptive management.

## Conceptual framework

It has been increasingly recognized that the complex characteristics of health systems—ambiguity, unpredictability and path dependency—make the blueprint approach to scaling-up difficult (Gilson and Schneider, 2010; Husain, 2017). As a result, scaling-up innovations is more than the expansion of the innovations per se, but requires a great deal of strategic planning, managerial flexibility and ‘learning by doing’ pragmatism (Paina and Peters, 2012). As the WHO and some think tanks repeatedly emphasize, *‘[s]trategic planning for the expansion and institutionalization of successfully tested health systems innovations is essential, but often does not happen… As a consequence, planning remains* ad hoc *and is often limited to statements about broad goals and the extent of scaling-up that is to be accomplished…[therefore], [a]ttention to scaling-up requires systematic planning of how pilot-tested innovations can be implemented on a larger scale and achieve broad impact.’* (Simmons *et al.*, 2007; WHO and ExpandNet, 2010).

This paper argues that an experimental approach, denoted as ‘scaling-up through piloting’, serves as a strategic pathway for complex health policy reform in a large country. Hailed as a cornerstone of evidence-based policymaking, piloting allows the government to test the innovation at stake in real-world settings at small and controlled scales, hence reducing the risk of potential failure or negative side effects (Jowell, 2003; Nair and Howlett, 2016). Yet, the old conception of piloting merely as a device for trial-and-error testing of new ideas has proved to be too simplistic, as piloting is increasingly seen as a versatile governance tool that can serve multiple purposes, such as demonstration and early implementation (Ettelt *et al.*, 2015).

**Figure 1.**
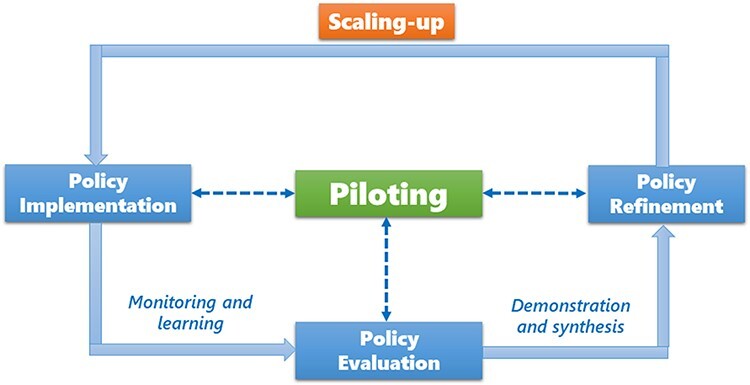
Scaling-up through piloting: a conceptual framework.

Source: the author

Illustrated in Figure 1, the conceptual framework of this study recognizes the crucial role of piloting in scaling-up that is seen as a strategic policy process. Theoretically, pilots occupy a space somewhere between policy formulation and implementation (Ettelt *et al.*, 2022). While the ultimate purpose is to inform the formulation of national or regional policies with locally derived evidence, piloting also constitutes part of the formal implementation process at the local level (Jowell, 2003; Ettelt *et al.*, 2015). In the meantime, rigorous evaluation of piloting results is always needed to facilitate national-level policy refinement. As a result, piloting connects at least three stages of the policy cycle: formulation (refinement), implementation and evaluation. It is therefore suggested that a ‘feedback loop’ is pivotal to piloting, and implementation should not be taken as a one-off but as an iterative process (Jowell, 2003).

This conceptual framework underscores the centrality of feedback loops in the scaling-up process. ‘Off the shelf’ diffusion is generally not seen as an appropriate strategy and there is a broad agreement that scaling-up must adapt the innovation to the local context, incentives and institutions, anticipating unintended consequences (Simmons *et al.*, 2007; Paina and Peters, 2012). Therefore, close monitoring and recursive learning are essential. Scaling-up through piloting is characterized by three distinctive features.

- An experimental approach to scaling-up that recognizes ‘one size doesn’t fit all’, so multiple innovations may be piloted to allow maximum testing and adaptation across geographic and institutional circumstances.
- Early implementation combined with proper evaluation of piloting informs policy refinement through an iterative process.
- Various policy learning mechanisms facilitate piloting and accelerate scaling-up to a wider scope.

## Research background

The importance of provider payment mechanisms is widely known in the health policy community. Among the handful of mechanisms prescribed by theory, people know that each of them creates distinctive incentives and signals influencing the behaviour of service providers (Ellis and McGuire, 1990). There are deep tradeoffs between alternative methods, as no payment mechanism is able to simultaneously balance cost containment, quantity of supply and quality of service in a perfect way (Barnum *et al.*, 1995). In reality, however, there has been a global trend to move away from retrospective payment mechanisms, especially FFS, towards prospective alternatives such as capitation, global budget and case-based methods. The driving forces behind such change include FFS’s inbuilt cost-inflationary inclination and the mounting pressure for cost containment in most health systems (Tan and Melendez-Torres, 2018). The past two decades have witnessed a rapid scaling-up of these payment innovations worldwide, particularly the rapid diffusion of DRGs in both the global south and the global north (Annear *et al.*, 2018; Mathauer and Wittenbecher, 2013).

Underpinned by patient classification systems primarily in hospital settings, DRGs categorize services provided to patients according to principal and secondary diagnoses, patient age and sex, the presence of co-morbidities and complications, and the procedures performed (Mathauer and Wittenbecher, 2013). Each DRG then constitutes a category for a predefined level of monetary payment based on a homogenous resource consumption pattern, regardless of the specific circumstance of each case (Annear and Xu, 2015). First introduced in the USA in 1983 as part of the prospective payment system for hospitals under the Medicare programme to control hospital spending, DRGs seek to address the shortcomings of FFS in hospital payment that create incentives for oversupply of services. As a prospective payment mechanism, the DRG model shifts the financial risk to hospitals and encourages efficient use of medical resources (Annear and Xu, 2015).

Despite these significant potentials, however, the scaling-up of DRGs is associated with critical challenges. First, reforming provider payment methods is arguably one of the most formidable tasks in a health system. The tremendous technical complexities alone would deter policymakers away from such attempts, particularly in countries where policy capacity is low and the political environment is volatile (Mathauer and Wittenbecher 2013; Bali, 2020; Abiiro *et al.*, 2021). More importantly, payment reforms are often associated with enormous uncertainties when put into practice. DRGs, for example, impose substantial pressure for behavioural change on hospitals and physicians and may erode their financial interest and professional autonomy. Consequently, numerous studies have shown the unintended outcomes resulting from inadequately managed scaling-up of such new payment mechanisms (Chiang *et al.*, 2015; Jian *et al.*, 2015). Common consequences associated with DRGs include opportunistic increases in hospital admissions, intentional and wrongful ‘up-coding’ and under-provision of necessary services (Mathauer and Wittenbecher, 2013; Tan and Melendez-Torres, 2018).

Health governance in China is characterized by a ‘dual steward’ structure in which the National Health Commission and its local arms regulate both public and private health facilities, while the local Healthcare Security Administrations (HSAs), which manage social health insurance (SHI) funds, are the third-party purchasers of health services (He *et al.*, 2022). The HSAs enjoy broad powers in health financing, including that of setting provider payment rules. Healthcare in China is mainly financed through two major SHI programmes that collectively cover the vast majority of people. In its decentralized health system, the majority of public hospitals—the key providers of health services in China—are owned by various levels of local government while SHI programmes are predominantly managed at the prefectural level, forming a rather fragmented governance structure. Although the central ministries make national policies, these policies have to be adapted locally. Given the wide interregional disparities in socioeconomic status and fiscal capacity, significant policy discretion is granted to local governments in health affairs (Ramesh *et al.*, 2014).

As extensively documented in the literature, a multitude of misaligned incentives drove Chinese hospitals and physicians towards vast provision of unnecessary care, leading to a double-digit escalation of health expenditure for more than two decades (Eggleston *et al.*, 2008). People’s financial accessibility to healthcare was significantly weakened until the 2000s. As the dominant mechanism for paying providers, FFS had long been recognized as a key cause of galloping cost inflation in China (Hu *et al.*, 2008; Yip *et al.*, 2010). The move away from FFS was advocated for a long time, but this yielded limited outcomes owing to the weak technical capacity of governments and hospitals (Meng, 2005; Gu and Page-Jarrett, 2018). Local initiatives—especially case-based methods—started to emerge in the late 1990s; some were initiated by hospitals in order to attract more patients and improve service efficiency, while others were pilot programmes introduced by local health departments or SHI offices primarily for the purpose of cost containment (Du, 2007; World Bank, 2010).

Preparation for case-based payment reform dates back to the early 1990s when the city of Beijing developed a preliminary disease classification system based on its local inpatient profiles that leaned on the standards of the US All Patient DRGs system. The Ministry of Health (MoH) launched an initiative in 1992 to develop quality standards for 102 diseases in Chinese hospitals, which became the foundation for case-based payment in China (Müller and Ten Brink, 2021). In 2004, the MoH started a loosely organized pilot programme and selected seven provinces to try out case-based innovations. Social learning was accelerated with the infusion of international experience offered by key external organizations, particularly the World Bank (Müller and Ten Brink, 2021).

Notwithstanding commendable progress, however, case-based payment reforms in this period were associated with three major weaknesses. First, the coverage of diseases was rather small, in part due to the complexities of disease classification. Hospitals were found to game the system by shifting costs away from the case-based payment system to FFS when the costs exceeded the payment limit (World Bank, 2010). Second, many local initiatives were merely single-disease price caps in nature rather than DRGs. Except for a few localities where hospitals tied case-based payment rates to standard treatment protocols, most hospitals adopted a very rudimentary method by simply averaging past medical expenditures on specific diseases (Meng, 2005; Du, 2007). Third, motivated primarily by cost containment concerns, these local initiatives paid little—if not no—attention to quality assurance of medical services. There is limited evidence that the new payment mechanisms improved the quality of services (World Bank, 2010).

## Methods

This study draws on both primary and secondary data. Policy documents, scholarly publications, official statistics and the grey literature pertinent to China’s health policy reform were first collected. These secondary data helped the author better understand the historical evolution, official policy framework and specific steps taken with regard to provider payment reforms. A total of 20 semi-structured in-depth interviews were performed with key stakeholders involved in the payment reform. Conducted between March and May 2022, these interviews sought to understand the reform programme from various stakeholders’ points-of-view. Ethical approval was granted by the author’s university. In view of travel restrictions, interviews were conducted in an online mode, with each taking ∼60 min. Interviewees were reassured of confidentiality and anonymized in this paper as per their request.

Recruited through purposive and snowball sampling, interviewees included 12 senior and mid-rank government officials, three policy advisors, three hospital managers and two think-tank researchers. Interviewees were based in Beijing, Shanghai, Guangdong, Fujian, Gansu, Shandong, Henan and Hubei. The institutions to which they were affiliated include the National Healthcare Security Administration, national and prefectural health commissions, provincial and prefectural HSAs, public hospitals and research institutions. Interviews with government officials intended to comprehend: (1) the rationale and programming of the reform; (2) its progress and challenges encountered; and (3) the institutional mechanisms underlying the pilot programme. Interviews with other stakeholders sought to capture the operational dynamics of the payment reform through their own participation. When ambiguities or disagreement emerged from interviews, they were resolved immediately through follow-up questions.

Transcripts were made through typing and handwriting and were immediately transcribed verbatim into Chinese. Qualitative data collected from primary (interviews) and secondary sources underwent three rounds of corroboration in order to accurately understand the policy vision and its actual implementation. Open coding and axial coding were performed to generate key conceptual themes that neatly fell into the three sub-categories to be presented in the next section.

## Results

### Pilot design

The previous local trial-and-error efforts yielded useful lessons for policymakers who officially announced in 2017 that the ultimate goal was to build a ‘hybrid provider payment system dominated by case-based mechanisms’. The main model conceived then was DRGs, and the State Council was planning to launch a pilot programme to test it out under a unified national protocol. The initial trialling, however, encountered considerable difficulties. First, the crucial capacities necessary for full-scale application of DRGs were not present in many regions. The majority of hospitals by then had not built up an adequate electronic medical record system that is essential for DRGs. A research report suggests that the penetration rate of electronic medical records in Chinese hospitals was <40% by 2018 (Chen and Fang, 2021). The implementation of DRGs innately requires high managerial capacity in terms of cost accounting, behavioural adjustment for frontline physicians and strict compliance with clinical pathways. Most hospitals were found not ready for its full implementation.^1^

Second, a DRG-based payment system encourages efficient use of resources because overutilization leads to financial loss while payment surplus becomes net revenues. However, hospitals in China have virtually no power of adjusting the fee schedule to reflect their actual costs and use of resources. The prices of pharmaceuticals and consumables, which account for the lion’s share in a hospital’s cost structure, remain rigidly regulated by the government (He *et al.*, 2022). This peculiar situation essentially means that hospitals had little room to adjust their behaviours to strike a balance between containing costs and earning revenues. Furthermore, anecdotal materials as well as the interviews suggest that the initial DRG classification was associated with considerable flaws, which were questioned by frontline physicians (Wang, 2019).^2^

An interim evaluation of the DRG pilot in 2020 found that merely 8 cities (out of 30) had met essential technical requirements and 8 other cities were rated unsatisfactory. It became clear to policymakers that the conditions for full implementation of DRGs were not present (China Health Insurance, 2020). At this juncture, DIP, an alternative case-based payment mechanism, was co-opted by policymakers. Emerging from local experiments mainly in Guangdong Province, DIP represented a less technically demanding approach to case-based payment and was considered more scalable. Table 1 below compares the two models.

**Table 1.** A comparison of DRG and DIP^a^ in China. Source: summarized by the author, with reference to Qian *et al*. (2021) and Lai *et al*. (2022)

	DRG	DIP
Patient classification rules	Develop 26 major diagnostic categories (MDCs)→divide each MDC into medical and surgical categories based on principal diagnosis or surgical procedure codes→define DRGs by considering patient characteristics, complications and comorbidities	Combinations of principal diagnosis ICD-10 codes and procedure ICD-9 CM codes^b^. Demographic factors not considered.
Number of groups	Hundreds (<1000)	≈13 000
Number of principal diagnoses in a group	Multiple	One for most groups
Data quality requirements	Higher than DIP	Lower than DRG
Payment modality	Payment rate for each case determined ex ante; total amount of payment capped by regional global budget.	Each group assigned with a certain number of ‘points’ reflecting its relative usage of resources; monetary value of each point determined ex post by regional global budget and city-wide point sum.

a DIP practice varies across localities. The information presented in this table is mainly based on the practice of Guangzhou, one of the pioneer cities of the DIP innovation.

b ICD-10: International Classification of Diseases (10th edition). ICD-9 CM3: International Classification of Diseases (9th edition) Clinical Modifications.

Based on the patient classification approach, both models are designed for paying inpatient services, but there are major differences between them. Using a clustering approach to classify patients according to direct combinations of principal diagnosis codes and procedure codes, the DIP system develops a larger number of classification groups (∼13 000 in Guangzhou, for example). Different from the DRG model, DIP classification does not consider demographic factors such as age or sex (Qian *et al.*, 2021; Lai *et al.*, 2022). Each DIP group is assigned a certain number of ‘points’ that reflect the citywide resource utilization relative to different groups. Importantly, the assignment of points also considers the global budget in order not to cause any financial risk to the SHI fund. While the relative points assigned to each DIP group are fixed, the actual monetary payments made to hospitals are determined *ex post* based on the point value that is pegged to the global insurance budget. A DRG system is typically developed based on the analysis of massive amounts of data and sophisticated algorithms, whereas a DIP system is associated with lower requirements for the quantity and quality of data because the payment formula is largely set through analysing medical expenditure data of recent years.

**Figure 2.**
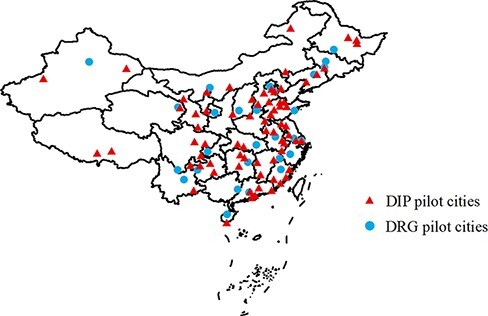
Geographic distribution of DRG/DIP pilots in China.

Source: drawn by the author based on government documents

Since 2020, China’s pilot programme in provider payment reform has been characterized by a dual-track arrangement, with both DRGs and DIP being piloted. Underpinned by the belief that ‘one size doesn’t fit all’, this arrangement gives due recognition to wide regional disparities in financial and managerial capacity of local health systems. Launched in 2019 and 2020 respectively, the DRG and DIP pilots have been undertaken on a semi-voluntary basis. Provincial authorities were required to recommend cities with necessary conditions and the National Healthcare Security Administration (NHSA) made the final selection. Local governments were given high autonomy in deciding which model to join. The final line-up consists of 30 DRG pilot cities and 71 DIP pilot cities (the geographic distribution is shown in Figure 2). A further analysis indicates that DRGs are adopted primarily in big cities (e.g. Beijing, Wuhan, Chongqing, etc.) or medium-sized ones with prior experience in running case-based models (e.g. Jinhua, Foshan, Wuxi, etc.), whereas DIP appears to be more popular among smaller cities. Policymakers made sure that at least one city was enlisted from each province, thus paving the way for local adaptation and scaling-up in the near future. Within each pilot city—except in Shanghai and Tianjin—only one payment mechanism may be chosen in order to avoid operational complications stemming from incompatibility issues.

The interviews suggest that this dual-track piloting design is intended to ‘play safe’ amidst high uncertainties. Dr Z, a senior official in the NHSA, explained:

*They [DRGs and DIP] are currently being promoted in parallel. The key purpose of doing so is to minimize potential risks in payment because we don’t fully know to what extent each of them is effective. The cross-reference [of piloting practice] between the two also helps us define the payment rates in a more accurate and reasonable way. In the long run, the experiences gained through this dual-track piloting will give us more clues with regard to their future integration…Virtually no payment mechanism is perfect. Despite decades of DRGs practice abroad, its application in China inevitably confronts big challenges, in light of our peculiar conditions. There remains a question mark as to whether this ‘imported’ idea [DRGs] will work well in China. In comparison, DIP as a domestically developed payment mechanism accounts for these local conditions and may be easier to implement in the short run.* ^3^

In fulfilling the function of experimentation, however, the working of piloting rarely follows the strict experimental rule of randomization and controllability, which are oftentimes impractical and unsuited to reality (Jowell, 2003; Ko and Shin, 2017). Instead, policy piloting is found to serve multiple purposes, including experimentation, early implementation, demonstration and learning (Ettelt *et al.*, 2015). This study finds that the selection of pilots in China’s payment reform does not follow a comparison-control group line of thinking. The selection criteria set by the policymakers focused on ‘capacity and preparedness’. A member of a local expert panel rightly pointed out the logic: *‘Nobody [policymakers] wants a pilot to fail. As a result, the central ministries normally choose the localities with better conditions that are more likely to succeed [in the pilot]’.*^4^ Multiple interviews suggest that firm support from local government, strong capacity in both administration and big data analytics and good track record were key considerations.^5^ The NHSA explicitly stipulated that pilot cities must have a sufficient surplus in their SHI fund, reflecting the ‘play safe’ mentality in light of the potential financial risks associated with trying new payment methods.

Within provinces, local officials put greater emphasis on ‘representativeness’ when selecting pilots. They are certainly aware that full implementation of case-based methods will start soon, and therefore, understanding ‘what works in what context’ is crucial for provincial governments at the piloting stage. Take Hubei Province in central China, for example. Its line-up in the piloting programme well illustrates the pursuit of representativeness. Among the three pilot cities, Wuhan is the provincial capital and the biggest medical hub in the entire central economic belt. With a developed medical system and strong administrative capacity, Wuhan was chosen to pilot DRGs. Selected for the DIP pilot, Yichang represents ordinary prefectural cities while Jingzhou is representative of cities encountering specific demographic challenges.^6^

It must be stressed that the dual-track design does not mean that the DRG and DIP pilots are undertaken in systemic isolation. Shanghai and Tianjin were exceptionally designated as the two cities where both models were to be piloted. Central and local policymakers required tertiary hospitals to test DRGs while DIP was to be tried in lower-tier facilities. The purpose of doing so was to gain in-depth contextual knowledge on the comparative strengths and weaknesses of the two models. Although DRGs and DIP are being tested in different levels of facilities, the ‘cross-reference’ in the same city still offers policymakers a quasi-lab setting to deliberate as to how the two payment models can possibly be integrated, with city-level factors being held constant.^7^

### Early implementation

Although the dual-track design reflects policymakers’ uncertainty about the universal scalability of DRGs, the strategic direction towards a case-based payment regime is set in stone. The piloting, as a result, is tasked with a ‘road test’ mission to support future policy integration and refinement. In this sense, testing is combined with early implementation in this piloting programme. Mr C, a key official involved in the national piloting programme, explains:

*SHI policies in China have passed the stage of ‘letting a hundred flowers blossom’. We now have a ‘top-level’ design to guide the reforms. In the past, all sorts of local trial-and-error experiments were undertaken in a bottom-up way, and we’ve learned a lot [from them]. It’s now time to do it top-down: the policy framework and major institutions have been established, and what to do next is to implement them…Yet, national policies are broad in nature and local adaptation is necessary before full implementation. Through piloting, we [central policymakers] need to know how DRGs and DIP perform on the ground. We hope that good local experiences can be absorbed to inform national-level policy adjustment, and refined [payment] policies can in turn be used to guide local practice. This would be a healthy cycle.* ^8^

Policy piloting is a useful governance tool for gaining necessary evidence and knowledge for policymaking (Nair and Howlett, 2016). Key to the generation of such evidence and knowledge are constant monitoring and productive learning. In contrast to the early attempts at payment reform in the past decade, both policymakers and local implementers are now in a much stronger position to carry out this current ambitious programme because of the impressive strides made in digital infrastructure and analytical capabilities (He *et al.*, 2022). The NHSA now owns a highly powerful technological system—described as an ‘information highway’—connected to not only all its local offices but almost every SHI-designated hospital.^9^ This big data platform enables policymakers to closely monitor local progress and retrieve data for learning and evaluation purposes. A very large number of experts was invited to form a nationwide advisory group to provide technical guidance to local implementers.

The fieldwork revealed multidirectional policy learning: (1) horizontal learning between local implementers, (2) vertical learning steered by central policymakers and (3) network-based social learning. First, local officials and hospital managers frequently engage in both informal exchange of ideas and formal learning such as field visits and seminars to share ‘best practices’. Second, the NHSA offers a variety of channels to facilitate peer learning among local implementers and to gain knowledge about the implementation of DRGs and DIP. These channels include all sorts of training programmes, sharing sessions, model demonstration conferences, onsite promotion meetings, policy brief series and monthly work digests. National-level work conferences and onsite promotion meetings appear to be particularly useful because these high-profile conferences are able to cement support from local leaders, which is essential for piloting.^10^ These conferences also serve as an ideal platform for ‘model demonstration’, where overachievers are invited to share their experiences with peer officials from all over the country, which is considered a high political honour in China.^11^ The third learning mechanism is network-based. Official think tanks, research societies and professional associations extensively participate in knowledge transfer, augmenting the power of learning to an unprecedented level. For instance, the first China CHS-DRG/DIP Reform Conference held in 2021 was attended by two million online participants, most of whom were local technocrats, hospital managers and even ordinary physicians. A hospital manager underscored:

*‘The (DRG/DIP) reform is a matter of bread and butter for all hospitals. Everybody is eager to learn!’* ^1^

### Evaluation, policy refinement and scaling-up

In principle, all piloting is associated with some form of evaluation (Ko and Shin, 2017; Ettelt *et al.*, 2022). As explained earlier, recent advancements in digital infrastructure have made it possible for policymakers to collect massive amounts of data to evaluate the pilots. To do so, the NHSA has devised a unique cross-provincial peer review scheme staffed by experienced technocrats and experts. Mr X explained the peer review process in a detailed manner:

*The NHSA has organized two rounds of peer review thus far. The first objective is to evaluate and learn and the second one is to promote the scaling-up. The NHSA plays the main role [in the evaluation] and colleagues from different regions join the team. Last year, we [Hubei Province] were evaluated by the panel from Jiangxi Province. There were division directors, experts, and representatives of their pilot cities in the panel. They visited all of the three pilot cities in our province and even inspected hospitals. They scrutinized our [DRG/DIP] case classifications, information infrastructure, and payment protocols, and audited the [payment] mock run. Later on, our [Hubei] panel was assigned by the NHSA to evaluate the pilots of Fujian Province in a similar way. We set fairly high standards [for the evaluation] and looked at lots of operational details in their new payment system. The evaluation combines both qualitative investigation and quantitative evidence, and it is conducted by professionals. We believe that the results are reliable.* *^12^*

Evaluation results lead to a series of performance ratings: excellent, good, pass and unsatisfactory. Good performers are subsequently established as national ‘demonstration sites’. Policymakers are keen to scale-up these experiences to the rest of the country. Delegations from other pilot sites frequently pay field visits to these exemplar cities, trying to learn the best practice. To date, 18 pilot cities have been designated as DRG demonstration sites and there are 12 demonstration sites for the DIP pilot. The evaluation is expected to generate necessary pressure for improvement through ‘naming and shaming’ effects.^13^ Four DIP pilot cities in the first round of evaluation were rated as unsatisfactory and were required to make significant efforts to catch up. For policymakers, evaluation is ultimately intended to accelerate peer learning and performance improvement.^14^ For local implementers, the pressure resulting from poor evaluation results is not inconsequential because *‘having a bad score not only makes you lose face” in front of peers from all over the country, but may also create an impression [to local leaders] of your incompetence or lack of effort that can be detrimental to your career’*.^15^ As such, concerns regarding organizational reputation and individual careers prompt local implementers to take the evaluation seriously.

Constant monitoring and formal evaluation have proved to be extremely valuable for policy synthesis and refinement. For instance, the classification list—the most crucial operational guideline for DRGs—has undergone two rounds of amendment, taking into account lessons learned from the pilots. Through this ‘road test’, policymakers also discovered a multitude of technical problems with regard to compatibility of information systems, quality of data and data processing protocols, among others, all of which were subsequently fixed. In April 2022, the NHSA launched a dedicated DRG/DIP e-module that was integrated into the nationwide SHI information system. Connected to all SHI offices and hospitals, this module standardizes DRG/DIP payment and data collection protocols while leaving local governments with sufficient autonomy for adaptation. This standardization has paved the way for scaling-up to the rest of China. Aside from the 101 centrally steered pilots, some provincial governments have also initiated their own pilot programmes. By mid-2022, there were >200 cities running pilots, accounting for two-thirds of prefectural cities in China. Scaling-up has thus been accelerated.

**Figure 3.**
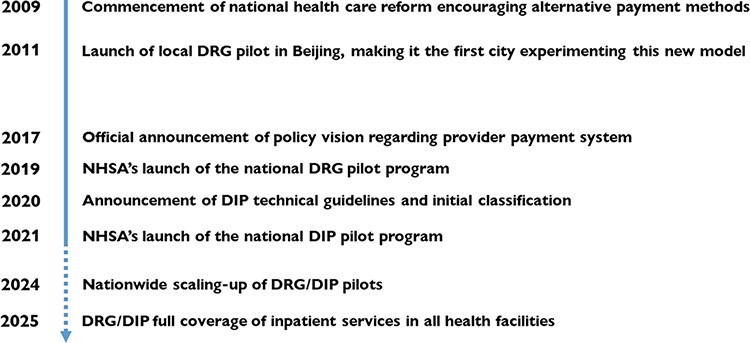
The chronicle of provider payment reforms since 2009.

Source: summarized by the author

Figure 3 illustrates the key milestones of the payment reform. In a newly announced ‘Third-Year Action Plan for DRG/DIP payment reform’, the central authorities envision that the twin payment mechanisms will be scaled-up to the entire country by the end of 2025. In the interim, piloting will be expanded to a larger scale between 2022 and 2024. Provincial governments are required to enlist at least 40, 30 and 30% of cities to try out DRG/DIP in 2022, 2023 and 2024, respectively, effectively scaling up the new methods within each province by the end of 2024. Ultimately, the vast majority of inpatient services should be paid through either of the twin methods by 2025. Although both senior interviewees from the NHSA suggested that DRGs and DIP may somehow be integrated in the future, there has been no such official indication to date. Scaling-up will proceed through piloting for a few more years.

## Discussion

This study has traced the development of China’s ongoing provider payment reforms and characterized a distinctive pathway denoted as scaling-up through piloting. The reform thus far well manifests China’s long tradition of ‘experimentation under hierarchy’ in which the central state sets a policy objective and steers local agents towards various forms of piloting activities to inform central policymaking (Heilmann, 2008). The original vision set in 2019 was to pilot DRGs and eventually scale it up, but it soon became clear that the project was much more complex than expected. The policymakers then demonstrated remarkable pragmatism and embarked on a dual-track piloting scheme on a much larger scale that essentially recruited one-third of cities nationwide. Scaling-up since then has been undertaken through continuous piloting, and this pathway has forged a phased implementation process, allowing new payment models to be tested, evaluated, compared and adjusted in a full spectrum of local contexts before being rolled out nationally. This phased implementation creates a ‘slower is faster’ effect (Paina and Peters, 2012), as it helps reduce long-term negative consequences arising from improperly managed scaling-up in a complex system.

Zooming out the China case to an international comparative perspective, one may find that the scaling-up of case-based payment mechanisms in China offers health policymakers of other middle-income countries useful lessons regarding how to manage complex scaling-up through an experimental approach. The international literature has repeatedly shown that many meaningful scaling-up endeavours in middle- and low- income countries yielded failures as a result of poor strategic planning and grave capacity deficit (Simmons *et al.*, 2007; Bali and Ramesh, 2021; Abiiro *et al.*, 2021). When innovative policy tools are introduced to the developing context, tremendous work is needed to recalibrate the tools at not only the macro level but also the micro level that requires developing innate technical skills and anticipatory capabilities (Bali, 2020). At the macro level, scaling-up through piloting offers policymakers necessary latitude for error detection and correction as well as tool recalibration that are essential for national policy refinement. At the micro level, this pathway is conducive to capacity building and skills transfer. Capacity here includes both analytical capacity and operational capacity (Husain *et al.*, 2021); both are vital for sustainable scaling-up efforts. Throughout the course of China’s provider payment reforms in the past years, the role of phenomenal improvement in both capacities has been very salient. In the meantime, multiple forms of training and technical assistance are provided to facilitate capacity building and skills transfer.

## Concluding remarks

In summary, China’s ongoing healthcare reform has managed to scale up the DRG and DIP innovations in a dual-track fashion through continuous piloting. This paper concludes that scaling-up through piloting represents a more robust and dynamic pathway of health sector reforms as compared to conventional policy processes in a complex system. Error detection and correction and recalibration of new policy tools can support national level policy refinement in the midst of deep complexities.

This paper draws three additional insights. First, in health systems of large countries or that face high levels of uncertainty, scaling-up innovations through piloting offers both policymakers and implementers great opportunities for learning by doing. Equally important, this pathway leaves stakeholders, especially health facilities and frontline staff, the requisite time to adapt innovations to local circumstances. Complex healthcare reforms could benefit significantly from a guided approach to scaling-up that combines strong central steering and local policy activism. As such, a dynamic structure of central–local relations operates as a conducive institutional factor because, ultimately, scaling-up through piloting is built on a feedback loop informing national-level policy formulation and refinement that incorporates new evidence and knowledge gained from local practice.

Second, the success of scaling-up relies heavily on policy learning. This study has noted a host of multidirectional learning mechanisms connecting central policymakers, local implementers, research institutions and industrial associations. Underlying this dynamic learning are what Husain (2017) describes as ‘informational devices’ and ‘informational infrastructure’ within the Chinese policy system. Scaling-up through piloting certainly requires some forms of evaluation, but the emphasis is on learning and improving pilot interventions instead of merely measuring their outcomes compared with the baseline (Ettelt *et al.*, 2022). In other words, evaluation for the sake of formality or legitimacy is of little value. Instead, ‘evaluation for monitoring’, ‘evaluation for improvement’ and ‘evaluation for refinement’ should be held as chief principles.

Third, I certainly acknowledge that some conditions discussed in this study are highly ‘Chinese’, such as strong central political authority. Indeed, experimentalism has been the typical process of policymaking, or, ‘policy style’ in China (Husain, 2017; Howlett and Tosun, 2018). The long tradition of experimental governance has forged dynamic central–local relations and a repertoire of policy instruments for the government to undertake scaling-up through repeated extensive piloting. Yet, this paper also argues that many aspects of the Chinese experiences discussed above are not particularly context-specific. Key principles of this pathway, especially recursive learning, simultaneous capacity building and managerial flexibility, are of critical value for policymakers in many other health systems.

## Endnotes

1. Mr H, Director, Office of Healthcare Reform, a major tertiary hospital in City Y, Shandong Province (Interview #4-11).

2. Mr F, Deputy Director, General Office, a major tertiary hospital in City Z, Henan Province (Interview #5-2).

3. Dr Z, Division Chief, National Healthcare Security Administration (Interview #4-2).

4. Dr L, member of expert panels of multiple provincial healthcare reforms in Guangdong (Interview #3-1).

5. Dr P, Former official, Ministry of Health (Interview #4-3); Dr C, Director, Healthcare Reform Office, D Provincial Health Commission (Interview #4-6); Prof. E, member of expert panels of multiple national and subnational healthcare reforms (Interview #4-4).

6. Mr X, Division Chief, Hubei Provincial Healthcare Security Administration (Interview #4-8).

7. Mr C, Division Chief, National Healthcare Security Administration (Interview #4-5).

8. Interview #4-2.

9. Interview #4-5.

10. Dr I, Director, Municipal Health Commission, City M (Interview #4-13).

11. Interview #4-3.

12. Interview #4-8.

13. Interview #4-2.

14. Interview #4-11.

15. Ms J, Deputy Division Chief, Municipal Healthcare Security Administration, City N (Interview #5-1).

## Reflexivity statement

This paper is a single-authored piece by a mid-career associate professor. Based in Hong Kong, he has been conducting research on China’s health care reform for more than a decade. A doctoral student and a research assistant (both are female) provided useful assistance.
